# Polymorphisms in the Human Aquaporin 4 Gene Are Associated With Schizophrenia in the Southern Chinese Han Population: A Case–Control Study

**DOI:** 10.3389/fpsyt.2020.00596

**Published:** 2020-06-26

**Authors:** Yung-Fu Wu, Huey-Kang Sytwu, For-Wey Lung

**Affiliations:** ^1^ Department of Psychiatry, Beitou Branch, Tri-Service General Hospital, National Defense Medical Center, Taipei, Taiwan; ^2^ Graduate Institute of Medical Science, National Defense Medical Center, Taipei, Taiwan; ^3^ Department of Microbiology and Immunology, National Defense Medical Center, Taipei, Taiwan; ^4^ Department of Psychiatry, Calo Psychiatric Center, Pingtung County, Taiwan

**Keywords:** schizophrenia (SCZ), single-nucleotide polymorphisms (SNPs), aquaporin 4 (AQP4), haplotype, astrocyte

## Abstract

**Background:**

In psychiatric illness, pathogenic role of neuroinflammation has been supported by multiple lines of evidence. Astrocytes contribute to the blood-brain barrier (BBB) with formation of the “glymphatic” drainage system of the central nervous system (CNS) through perivascular processes. Found primarily at the end-feet of astrocytes, the aquaporin 4 (AQP4) gene has been suspected to play putative roles in the development of psychiatric disorders as well as the clearance of the glymphatic system. However, there remain many uncertainties because of the limited research on AQP4. The present study is focused on the association between AQP4 gene polymorphisms and schizophrenia (SCZ) in the Southern Chinese Han population.

**Methods:**

Two hundred ninety-two patients and 100 healthy controls were enrolled in this study. To study the relationship of AQP4 gene polymorphisms and SCZ, genetic information was drawn from a cohort of 100 healthy controls and 100 matched patients with SCZ of Southern Han Chinese descent. Comparisons of the allele and genotype distributions between control and case groups were made using the χ2 test. Two-group comparisons were made to assess the linkage equilibrium and haplotype.

**Results:**

Three SNPs were found. In comparison to healthy controls, patients had higher T-allele frequencies at rs1058424 and G-allele frequencies at rs3763043 (p = 0.043 and p = 0.045, respectively). Furthermore, there is an association between the decreased risk of SCZ and the AA genotype at both rs1058424 (p = 0.021, OR = 2.04) and rs3763043 (p = 0.018, OR = 2.25) The TCG haplotype (p = 0.036) was associated with a potential risk of SCZ, while the ACA haplotype (p = 0.0007) was associated with a decreased risk of SCZ and retained statistical significance after Bonferroni correction (p = 0.006).

**Conclusions:**

An etiological reference for SCZ is provided by the association between AQP4 gene polymorphisms and SCZ in Southern Han Chinese population.

## Introduction

Both environmental and genetic factors play an important role in schizophrenia (SCZ), which is a multifactorial common psychiatric disorder (1–3). Given the lack of diagnostic neuropathology and defined biological markers for SCZ, it is hard to monitor the disease progression and treatment response. Currently, there is an increasing recognition in the significance of SCZ genetic polymorphisms, given the findings of genome-wide association studies (4). The modest increase in relative risk in SCZ is due to the cause by multiple genes.

However, more and more researchers have now identified the illness of SCZ is an inflammatory disease of the central nervous system (CNS) characterized by relapsing attacks of neuronal dysfunction (5). For more than 20 years, research has focused on the interactions of neurons and neuroglial cells, such as astrocytes and microglia, which result in neuroinflammation (6). Furthermore, they found that many aspects of disrupted neuronal and synaptic function, such as disrupted glutamate homeostasis, impaired action of antipsychotics, development of antipsychotic resistance, and increased permeability to inflammatory molecules may be related to the complex nature of the dysfunction of the blood-brain barrier (BBB) (7, 8). Clearance of waste for vertebrate CNS is carried out by the glymphatic system (9). The pathway consists of a para-arterial influx route for cerebrospinal fluid (CSF) to enter the brain parenchyma by BBB transport, coupled to a clearance mechanism for the removal of interstitial fluid (ISF) and extracellular solutes from the interstitial compartments of the brain and spinal cord (10). Initial arterial pulsation and regulation of exchange of solutes between ISF and CSF also depends on brain extracellular space contraction and expansion. Astrocytic aquaporin 4 (AQP4) water channels facilitate the convective bulk flow of ISF, which carries out clearance of waste products, excess extracellular fluid, and soluble proteins (11). The most abundant protein found in ependymal cell lining and in astrocytes in the ventricles showing the highest expression on perivascular astrocytes end feet surrounding blood vessels in CNS is the AQP4 water-channel protein (12). The AQP4 protein encoded by the AQP4 gene, which is located on chromosome 18, consists of five exons and four introns and, through an alternative splicing mechanism of the AQP4 protein, has two isoforms (13, 14). Coding regions of AQP4 are highly conserved, but noncoding regions demonstrate high sequence variation (15, 16). Coding nucleotide variants of AQP4 may influence conformational changes in protein structure, and this may alter the function of AQP4 proteins. Nevertheless, noncoding regions of the AQP4 gene are also thought to be involved in transcriptional and post-transcriptional regulation because of their binding affinity for transcriptional factors. In particular, the 3′ untranslated region (UTR) may be a target for microRNAs (miRNAs), which affect gene expression patterns (17). Theoretically, the total protein content and the pathogenesis of SCZ may be affected by the single nucleotide polymorphisms (SNPs) in the 5′ or 3′ UTRs of the AQP4 gene.

To summarize, due to its importance in maintenance of BBB structure, permeability, and structure, AQP4 may influence the glymphatic system in SCZ. It seems that AQP4 polymorphisms may play an important role in disease incidence, progression, and treatment response. However, most of the current gene-level studies on SCZ have not focused on the AQP4 gene. In our published studies (5), we have identified that differences in the treatment response in SCZ may be associated with the impacts of AQP4 variation. Furthermore, we have noted an association between biomarkers of treatment response and 3′ UTRs of AQP4 SNPs (rs1058424, rs335929, and rs376043) in patients with SCZ (18). We used a graphic method to dichotomize the two groups using selected cutoff points. For prediction of a higher severity of negative symptoms of SCZ, a log value of S100 calcium-binding protein B (S100B) level >1.78 may be sufficient. In conclusion, negative symptoms, poor control of neuroinflammation, and increased serum levels of S100B are more likely in patients with TAA haplotype of the AQP4 polymorphism. However, one limitation of this approach may be that in some instances the study outcome is altered when it is used instead of the traditional analysis in comparison with healthy individuals.

In this study, we further investigated the contribution of the AQP4 gene in our patient cohort by carrying out a matched case–control study. We aimed to investigate the relationship between the AQP4 polymorphisms and the risk of SCZ among the Southern Chinese Han population.

## Methods

### Study Participants

All patients were enrolled from the Department of Psychiatry of the Beitou Branch of Tri- Service General Hospital. Patients aged between 20 and 70 years who experienced SCZ between January 1, 2017 and December 31, 2019 were evaluated. The control group was drawn from the community. All the cases and controls were of Southern Han Chinese ethnicity. Written informed consent were collected from both healthy controls and patients in participation of this study, with approval from the independent ethics committee/institutional review board in Taiwan.

### DNA Extraction

Peripheral blood samples were provided by participants, using ethylenediaminetetraacetic acid (ETDA) tubes. DNA was extracted using an AccuBiomed iColumn 12 purification system and AccuPure Cell/Blood DNA Mini Kit (AccuBiomed, New Taipei City, Taiwan). First, 20 μl proteinase K and 200 μl whole blood were added to a 2-ml sample tube. The sample was spun down at 6,000 rpm for 30 s, and the tube was placed in the above system. Finally, the DNA samples were eluted at 100 μl elution buffer and the concentration are quantitate using Nanodrop. (ThermoScientific, Wilmington, Delaware, USA).

### Segment Selection and Primer Design

The entire sequence of the human AQP4 gene comprises the full-length human AQP4 gene plus 5 kb upstream and 2 kb downstream (21.3 kb in total). Data on the genetic variation of the entire gene were obtained from the HapMap project (http://hapmap.ncbi.nlm.nih.gov/) for 45 unrelated Chinese Han individuals in Beijing (CHB). The real-time hybridization method was used to analyze and detect the rs1058424 (T/A), rs335929 (C/A), and rs3763043 (G/A) SNPs (Roche Light Cycler 480II, Switzerland). The primer probes are listed in **Table 1**.

**Table 1:** List of primers/probes.

Hyb qPCR	Primer	Sequence (5′→3′)
rs1058424	Primer-Forward	GCAAGTGTCACTGCTCATCA
rs1058424	Primer-Reverse	AGGTGCCCTTATGATTTGGGA
rs335929	Primer-Forward	TCCCACATTACCTTGGGCAT
rs335929	Primer-Reverse	CCTTATGCATAGACTACCTTGGC
rs3763043	Primer-Forward	ACCGTGTGTCAAGATTTGGT
rs3763043	Primer-Reverse	TGAATGTGCATGACTGTGACA

### Polymerase Chain Reaction (PCR) Amplification

The PCR mix was prepared using a KAPA HRM FAST qPCR Kit (KAPA Biosystems, Wilmington, Massachusetts, USA). The reaction mixture contained 20 ng DNA, 1 µl 10 µM Forward and Reverse Primer, 2 µl 25 mM MgCl_2_, 10 µl 2× KAPA HRM FAST Master Mix, and PCR-grade water to a total of 20 µl. The PCR protocol began with pre-denaturation at 95°C for 120 s, followed by pre-denaturation at 95°C for 5 s and 60°C for 40 s, repeated for 45 cycles. After the PCR cycles, the melting-curve step was started at 95°C for 1 s and 35°C for 1 min. The melting curve was produced by increasing the temperature to 90°C and took a reading for every 0.14 s. Finally, the samples were cooled to 40°C for 1 min.

### DNA Sequencing, SNP Selection, and Genotyping

Salting-out method was used to extract genomic DNA from peripheral blood leukocytes. Based on the HapMap data for Han Chinese in the Beijing population, SNPs were tagged across the entire region of the AQP4 gene, selected using the tagger algorithm (http://www.broadinstitute.org/mpg/tagger/) with a pairwise approach, an r2 cutoff of 0.8, and a minor allele frequency >0.05. A total of three tag SNPs in the 3′ UTR (rs1058424, rs335929, and rs3763043) in two distinct gene regions were retrieved. TaqMan allele-specific discrimination assays on an ABI PRISM_7700 Sequence Detection System and analyzed with SDS software (Applied Biosystems, Foster City, CA, USA) was used for tag SNP genotyping. The characteristics of genotyped AQP4 tag SNPs are listed in **Table 2**.

**Table 2:** Characteristics of genotyped AQP4 tag SNPs.

rs number	Chromosome position	Distance from gene start	Gene position	Function	HWpval	MAF
rs1058424	24435545	3543	3′ UTR	Regulatory	1.186x10^-5^	A/T (0.428)
rs335929	24435587	3585	3′ UTR	Regulatory	0.549	A/C (0.492)
rs3763043	24435818	3816	3′ UTR	Regulatory	0.010	G/A (0.482)

*HWpval denotes p values for the Hardy–Weinberg equilibrium in the controls.

MAF, Minor allele frequency.

### Statistical Analysis

Statistical analyses were performed using SPSS Software 22.0 (IBM, Armonk, NY, USA). The case and control groups were identified using propensity score matching methods.

Continuous variables were expressed as mean ± SD. Categorical variables were expressed as N (%). Odds ratios (ORs) and 95% confidence intervals (CIs) were calculated. Patients were genotyped for risk SNPs to investigate the difference between groups. Allele and genotype frequencies were found by calculation of the Hardy–Weinberg equilibrium (HWE) of the three SNPs. A goodness-of-fit χ2 test was used to detect the HWE, and the Pearson χ2 test was used to compare allele distributions. Haploview 4.2 software (Broad Institute, Cambridge, MA, USA) was used to visualize linkage disequilibrium (LD) structure and haplotype (19). Haplotype frequency comparisons under different modes of hereditary were conducted using R package haplo.stats (20). Statistical analysis of haplotypes with traits and covariates was performed when the linkage phase was ambiguous. The χ2 test was used to assess the associations between alleles, genotypes, haplotypes, and the risk of SCZ, respectively. The Bonferroni correction was used in multiple independent comparisons (p < 0.00625 was statistically significant) to control for type I error, and the p-value was divided by the total number of loci or haplotypes.

## Results

### Patient Demographics and Clinical Features

We recruited 292 consecutive cases and 100 healthy controls. For the association study, the 100 cases were matched for age, sex, and education to 100 of the healthy controls. The demographic data for the cases and controls are listed in **Table 3**. There were 41 men (41.0%) and 59 women (59.0%) in the matched case group, and 48 men (48.0%) and 52 women (52.0%) in the control group. The mean age of the cases was 45.99 ± 23.26 years and that of the controls was 42.38 ± 13.11 years. The mean age at diagnosis of SCZ was 27.43 ± 9.31 years and the mean duration of illness was 18.56 ± 11.48 years.

**Table 3:** Clinical and demographic information on the participants, and baseline statistical analysis of the groups.

Variables	Case group (*n* = 100)		Control group (*n* = 100)		*p* value
	Number	%	Number	%	
Male	41	41	48	48	0.319
Age distribution (years)					0.374
20–29	13	13	18	18	
30–39	20	20	29	29	
40–49	26	26	22	22	
50–59	25	25	18	18	
60–69	16	16	13	13	
Educational level (years)					0.710
< 6	2	2	2	2	
7–9	0	0	1	1	
10–12	27	27	23	23	
> 13	71	71	74	74	
	Mean	SD	Mean	SD	*p*-Value
Age	45.99	23.26	42.38	13.11	0.658
Age at diagnosis of SCZ (years)	27.43	9.31			
Duration of illness (years)	18.56	11.48			

SCZ, schizophrenia.

### Allele and Genotype Analysis

We detected three SNPs (rs1058424, rs3763043, and rs335929) through the analysis of sequencing results. Allele frequencies and genotype frequencies of the detected SNP loci are listed in **Table 4**. When compared with two groups, we found that the patients had higher frequencies of the T-allele at rs1058424 and the G-allele at rs3763043 than healthy controls had (p = 0.043 and p = 0.045, respectively). In addition, the AA genotype at both rs1058424 (P = 0.021, OR=2.04) and rs3763043 (P = 0.018, OR=2.25) was associated with lower frequencies and a lower risk of SCZ when compared with healthy controls.

**Table 4:** Genotype distribution and allele frequencies of AQP4 SNPs between groups.

AQP4 tag SNPs	Case group (*n* = 100)		Healthy group (*n* = 100)		χ^2^	OR	95% CI	*p* value
	No.	%	No.	%				
**rs1058424**								0.041*
**Genotype**								
AT	40	40.0	27	27.0				
TT	28	28.0	24	24.0				
AT+TT	68	68.0	51	51.0				
AA	32	32.0	49	49.0	5.996	2.04	1.149–3.627	0.021*
**Allele**								
T	96	48.0	75	37.5	4.505	1.28	1.017–1.611	0.043*
A	104	52.0	125	62.5				
**rs335929**								0.306
**Genotype**								
AC	52	52.0	42	42.0				
AA	26	26.0	28	28.0				
AA+AC	78	78.0	70	70.0				
CC	22	22.0	30	30.0	1.663	1.52	0.803–2.875	0.259
**Allele**								
A	105	52.5	98	49.0	0.490	1.07	0.883–1.300	0.549
C	95	47.5	102	51.0				
**rs3763043**								0.036*
**Genotype**								
AG	46	46.0	34	34.0				
GG	34	34.0	30	30.0				
AG+GG	80	80.0	64	64.0				
AA	20	20.0	36	36.0	6.349	2.25	1.189–4.258	0.018*
**Allele**								
A	86	43.0	107	53.5	4.415	0.80	0.655–0.987	0.045*
G	114	57.0	93	46.5				

*< 0.05, **< 0.01, ***< 0.001.

### Linkage Disequilibrium and Haplotypes

**Figure 1:**
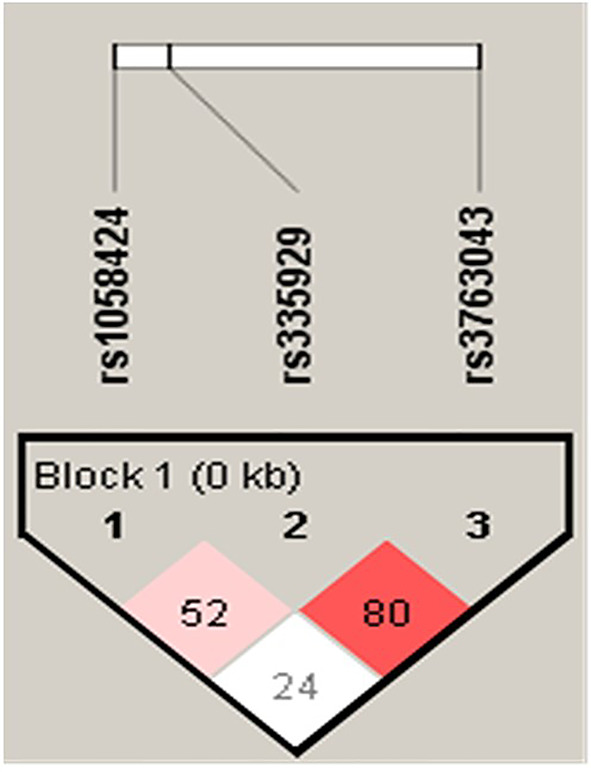
Linkage disequilibrium (LD) in AQP4 markers (rs1058424, rs335929, and rs3763043) in SCZ and controls in a population.

Each of the result of the LD and the relationship of haplotype distribution are showed in **Figure 1** and **Table 5**. Based on proximity of SNPs, prevalence of more than 1%, and LD, eight haplotypes were selected by authors. Global significance of the positive results were obtained using permutations (n = 100,000). The TCG haplotype (p = 0.036) was associated with a potential risk of SCZ, while the ACA haplotype (p = 0.0007) was associated with a decreased risk of SCZ and retained statistical significance after Bonferroni correction (p = 0.006).

**Table 5:** Predicted haplotypes from the AQP4 tag SNPs (rs1058424, rs335929, and rs376043) between 100 cases and 100 healthy controls.

Haplotype	Frequency	Case ratio	Control ratio	χ^2^	*p* value	Adjusted *p* value^a^
TCG	0.185	45.2: 154.8	28.9: 171.1	4.418	0.036*	0.288
AAA	0.171	32.7: 167.3	35.5: 164.5	0.142	0.706	1.000
AAG	0.165	35.6: 164.4	30.3: 169.7	0.499	0.480	1.000
ACA	0.127	14.2: 185.8	36.6: 163.4	11.38	0.0007***	0.006**
TAA	0.115	25.0: 175.0	20.8: 179.2	0.437	0.508	1.000
ACG	0.110	21.6: 178.4	22.5: 177.5	0.023	0.880	1.000
TCA	0.070	14.1: 185.9	14.0: 186.0	0.000	0.987	1.000
TAG	0.057	11.7: 188.3	11.3: 188.7	0.007	0.935	1.000

*< 0.05, **< 0.01, ***< 0.001.

a P values of no more than 0.00625 were considered statistically significant due to the Bonferroni correction.

## Discussion

Genetic factors play important roles in SCZ pathogenesis as indicated by epidemiological data (1). The genetic basis for SCZ may not be related to a single genetic variant but may be influenced by multiple genes acting synergistically with environmental factors to increase the likelihood of the disease developing. Genetic association studies offer a powerful approach for identifying the multiple and sometimes minor variables that modulate susceptibility to this common but complex disease. Despite the critical role of AQP4 in the pathogenesis of SCZ, polymorphisms in coding regions of the AQP4 gene are unlikely to confer a risk of SCZ. On the other hand, the noncoding regions of this gene showed considerable variation among various ethnic groups. We have noted that the 3′ UTR of the AQP4 gene locus presents a high degree of variation, with several polymorphic sites that may influence SCZ susceptibility in the Southern Chinese Han population (18). In this study, we confirmed the importance of these three SNPs in the 3′ UTR region and identified the possible risk and protective haplotypes in SCZ.

As we know, the CNS is the only organ of the body that lacks a traditional lymphatic system, and as a result, has developed unique adaptations for achieving fluid balance and interstitial waste removal. AQP4 channels, which are highly concentrated in astrocytes and ependymal cells lining in the ventricles, are the most common water channels in the CNS (21–23). Also, AQP4 is involved in BBB development, function, and integrity (24). Apart from its function in water homeostasis, many studies have shown possible inter-relations between AQP4 and the glymphatic system (25). Due to its particularly high expression at the BBB and blood CSF barrier, we try to emphasize that the genetic variants of AQP4 will modulate the bidirectional fluid exchange, the subsequent activation of microglia, the development of neuroinflammation, and the treatment response of therapeutics (5, 26).

AQP4 has been implicated in the pathophysiology of many neurological (23, 27–32) and psychiatric (33–35) diseases. In a previous study, AQP4 gene polymorphisms were examined in children with febrile seizures and revealed no association with the rs1058424 AT genotype or rs3763043 CT genotype SNPs. Furthermore, the two selected SNPs have been researched in relation to other neurological disorders. Burfeind et al. (36) reported that rs3763043 was associated with a more rapid cognitive decline after diagnosis of Alzheimer’s disease. Another study, involving patients with traumatic brain injury, revealed that the rs3763043 TT genotype was significantly more prevalent in those with poor outcomes (37). Heuser et al. (38) researched the AQP4 and Kir 4.1 genes in patients with temporal lobe epilepsy, and showed that rs1058424 was associated with the disease. Related studies have strongly pointed out the importance of water channels in the occurrence of neurological diseases, but the findings cannot be directly applied to psychiatric diseases that also originate from the brain.

Polymorphisms of the AQP4 gene have not been studied intensively in psychiatric diseases, possibly because of the highly conserved nature of the coding region. Studies in this area have been insufficient because AQP4 gene polymorphisms, especially in noncoding regions, vary among different populations. In the existing literature discussion, studies from Japan (39) and Taiwan (5, 18) have reported inconsistent results regarding the role of the AQP4 gene in SCZ. We found that Japanese research as early as 2005 was particularly focused on AQP4 SNPs (39). Although the results showed no direct association with rs3763043 in Japanese patients, it appears to be the earliest study of the correlation between SCZ and water channels. In an article published by our research team in 2012 (5), we hypothesized that the treatment response may be due to different genetic performance among individuals and the effect on changes in the function of water channels. Regarding the differences in the clinical findings related to drugs and clinical treatment effects, it seems that rs335929 may have an effect in influencing the dosage of antipsychotics. Both studies verified the importance of AQP4, but further discussion of the effect of AQP4 haplotypes was lacking. Our current study performed an analysis of eight haplotypes and showed the association of TAA haplotype and treatment response (18). We also noted that TCG haplotype was the most frequent haplotype while the ACA haplotype was the lowest frequent haplotype among 190 patients. In this case-control study, the TCG haplotype still had the highest frequencies in case group. However, the ACA haplotype was identified to be the most frequent haplotype in healthy control group. The significance of these two studies indicated that the 3′ UTRs of the AQP4 gene may be potentially associated with the incidence, progression and treatment response of SCZ in the Southern Chinese Han population.

Our study had some limitations. First, it was noted that patients with early onset of age and lower education levels were over-represented in the study. The majority of our 292 participants with SCZ had the characteristics of early onset of illness (age 25.30 vs. 27.43 years) and lower educational level (74.1% vs. 29.0% below 12 years) when compared with the 100 matched SCZ cases. Second, the current study had a small sample of healthy controls and a limited number of haplotypes (eight). Large numbers of cases and controls will be needed to increase the recombination of LD blocks and confirm our findings. Third, the Berkson bias, which is a kind of selection bias, may have existed, although the case group contained only patients with a diagnosis of SCZ. Most of the participants in this study had been hospitalized more than once and had received different medications for a long time before joining the study. Inclusion of patients with repeated hospitalizations was unavoidable. In addition, patients who could not cooperate with the treatment or for whom the treatment response was not good may have been transferred to another hospital and not enrolled in the study. Therefore, it was not possible to show the results for patients who have been diagnosed with SCZ for the first time and those who have not taken their medication regularly.

To our knowledge, this is the first case-control study to report the associations between these three SNPs and SCZ in the Southern Chinese Han population. We conclude that patients had higher frequencies of the T-allele at rs1058424 and G-allele at rs3763043 than healthy controls had. Furthermore, the AA genotype at both rs1058424 and rs3763043 was associated with a decreased risk of SCZ. The TCG haplotype was associated with a potential risk of SCZ, while the ACA haplotype was associated with a decreased risk of SCZ and retained statistical significance after Bonferroni correction.

## Data Availability Statement

The datasets generated for this study are available on request to the corresponding authors.

## Ethics Statement

The studies involving human participants were reviewed and approved by Institutional Review Board of Tri-Service General Hospital, Taiwan. The patients/participants provided their written informed consent to participate in this study.

## Author Contributions

Y-FW, with help of H-KS and F-WL, planned the present study’s content and analysis, interpreted the data and wrote the paper. Y-FW, H-KS, and F-WL initiated and performed the whole survey, analyzed the data and helped to interpret the findings and to write the paper. All authors contributed to the article and approved the submitted version.

## Conflict of Interest

The authors declare that the research was conducted in the absence of any commercial or financial relationships that could be construed as a potential conflict of interest.
